# Defining Golden Batches in Biomanufacturing Processes From Internal Metabolic Activity to Detect Process Changes That May Affect Product Quality

**DOI:** 10.1002/bit.28873

**Published:** 2024-10-27

**Authors:** Xin Bush, Erica J. Fratz‐Berilla, Casey L. Kohnhorst, Roberta King, Cyrus Agarabi, David N. Powers, Nicholas Trunfio

**Affiliations:** ^1^ U.S. Food and Drug Administration, Center for Drug Evaluation and Research, Office of Pharmaceutical Quality, Office of Pharmaceutical Quality Research, Division Pharmaceutical Quality Research VI Silver Spring Maryland USA; ^2^ U.S. Food and Drug Administration, Center for Drug Evaluation and Research, Office of Pharmaceutical Quality, Office of Pharmaceutical Quality Research, Division Pharmaceutical Quality Research III Silver Spring Maryland USA; ^3^ Department of Biomedical and Pharmaceutical Sciences, College of Pharmacy University of Rhode Island Kingston Rhode Island USA; ^4^ U.S. Food and Drug Administration, Center for Drug Evaluation and Research, Office of Pharmaceutical Quality Research Immediate Office Silver Spring Maryland USA

**Keywords:** bioprocessing, constraint‐based systems biology, multivariate analysis

## Abstract

Cellular metabolism plays a role in the observed variability of a drug substance's Critical Quality Attributes (CQAs) made by biomanufacturing processes. Therefore, here we describe a new approach for monitoring biomanufacturing processes that measures a set of metabolic reaction rates (named Critical Metabolic Parameters (CMP) in addition to the macroscopic process conditions currently being used as Critical Process Parameters (CPP) for biomanufacturing. Constraint‐based systems biology models like Flux Balance Analysis (FBA) are used to estimate metabolic reaction rates, and metabolic rates are used as inputs for multivariate Batch Evolution Models (BEM). Metabolic activity was reproducible among batches and could be monitored to detect a deliberately induced macroscopic process shift (i.e., temperature change). The CMP approach has the potential to enable “golden batches” in biomanufacturing processes to be defined from the internal metabolic activity and to aid in detecting process changes that may impact the quality of the product. Overall, the data suggested that monitoring of metabolic activity has promise for biomanufacturing process control.

## 1 Introduction

Biomanufacturing processes use living cells to create a drug substance with therapeutic effects (Zhang, Sun, and Ma 2017). These therapeutic effects are governed by specific characteristics of the drug substance's molecular structure referred to as Critical Quality Attributes (CQAs) (Yu et al. 2014). For example, the activity of a monoclonal Antibody (mAb) produced in a Chinese Hamster Ovary (CHO) cell process is partially dictated by the glycans attached to the mAb because of the role they play in binding the drug to its target (Zhang, Luo, and Zhang 2016).

Statistical analysis methods can be used to assess the performance of a bioprocess. In particular, multivariate analysis techniques have become a popular tool for identifying Critical Process Parameters (CPPs) and establishing their time‐dependent acceptable ranges (Huang et al. 2009). This is because CPPs tend to be highly correlated with one another and multivariate analysis techniques like Principal Component Analysis (PCA) (Maruthamuthu et al. 2020) and Partial Least Squares regressions (PLS) (Azer et al. 2023; Zavala‐Ortiz et al. 2020; Zavala‐Ortiz et al. 2022) can be used to identify summary variables that model the correlation structure in the CPPs. For example, a single principal component can characterize the relationship between the concentration of nutrients like glucose that tend to decrease and the concentration of viable cells and waste byproducts like lactate that tend to increase over the same time interval (Rathore et al. 2015; Schwab et al. 2016; Sokolov et al. 2017a). Thus, with principal component analysis a set of fewer summary variables of CPPs can be monitored to ensure a process will create consistently high‐quality drug substance. Further, the summary variables are sensitive to subtle changes in the many correlated variables they are derived from (Destro and Barolo 2022); this allows for process changes to be detected earlier and could potentially allow for corrective action in some cases (Sokolov et al. 2017b). The time‐dependent acceptable ranges of these CPPs are referred to as the “golden batch” because a future process operated within these ranges is known a priori to produce in specification product by virtue of the way the process was operated.

Cellular metabolism is the causal reason for at least some, and possibly most, of the variability observed in a given drug substance's CQAs (Okeley et al. 2013). For example, nucleotide sugar metabolism in the Golgi (Berninsone and Hirschberg 2000) is responsible for creating and attaching glycans to the mAbs produced in CHO cell culture processes (Wong et al. 2010). Further, the correlation structures observed in the macroscopic CPPs are primarily the result of the impact that the macroscopic properties have on metabolism (Helgers, Schmidt, and Strube 2022). For example, glycolysis is the metabolic process that breaks down the nutrient glucose into the waste byproduct lactate to generate the Adenosine Triphosphate (ATP) (Hara and Kondo 2015) that cells breakdown for energy to fuel the production of more viable cells and protein (Buchsteiner et al. 2018).

Therefore, a soft sensor based on cellular metabolism is proposed as a process monitoring tool during the biomanufacturing process operation to support the understanding of metabolic processes affecting product quality changes. A set of Critical Metabolic Parameters (CMPs) could be monitored as additional process information analogous to the way that macroscopic CPPs (Yeo et al. 2020) are monitored with current PAT approaches. CMPs could provide a way to monitor the evolution of the metabolic processes that affect CQAs directly (Sommeregger et al. 2017) instead of indirectly monitoring their evolution based on macroscopic process conditions. Constraint‐based systems biology models, such as Flux Balance Analysis (FBA) (Sha et al. 2016; Sou et al. 2015) and Metabolic Flux Analysis (MFA) (Beyß et al. 2019; Quek et al. 2010), provide a way to estimate metabolic reaction rates and metabolite concentrations inside the average cell of a bioprocess. Constraint based systems biology models have been combined with multivariate analysis in the past to understand differences in cellular metabolism between batches operated with different process conditions (Reimonn et al. 2016). In this study, FBA was applied to model central carbon metabolism and used the resulting metabolic reaction rates as inputs to multivariate models. The results show that the metabolic activity was reproducible among batches and that process changes could be reproducibly detected from changes in the metabolic activity without measuring the macroscopic properties used to induce the process change.

## 2 Methods

**Figure 1:**
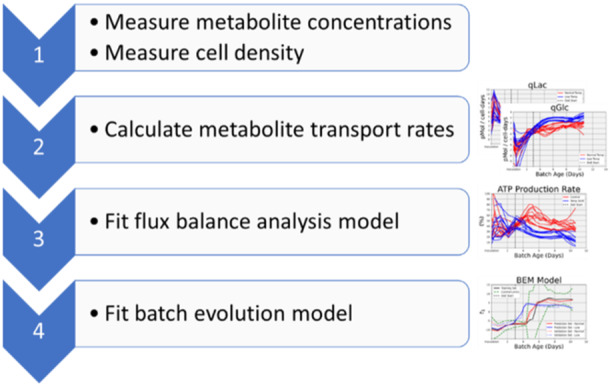
Flowchart for monitoring a biomanufacturing process from internal cellular metabolism.

A flowchart for the complete workflow is shown in Figure 1.

### 2.1 Cell Culture Process

In house CHO K1 cell line expressing an IgG1 for anti‐HIV‐1 (Teh et al. 2014) was cultured in Hyclone ActiPro media for production (Marlborough, MA). The cell cultures were inoculated at a density of 0.3 × 10^6^ cells/mL in a Sartorius Ambr 15 Cell Culture Workstation system (Göttingen, Germany). The 3‐day unfed batch phase was followed by a 7‐day fed‐batch phase. Hyclone Cell Boost 7a and 7b supplements (Marlborough, MA) were added daily once the glucose level dropped below 3.0 g/L to a target of 6.0 g/L. The DO was maintained at 50%, and the pH was maintained at 7.2 throughout all cultures. Normal operation of the cell culture process temperature was maintained at 37°C. To intentionally disrupt normal metabolic behavior, selected batches were reduced to 33°C at the same time of the first glucose feed and were held at this temperature for the remainder of the 7‐day process. A set of 24 batches were run for model training: 12 were operated at 37°C for the whole batch and 12 had their temperature reduced to 33°C on Day 3 at their first glucose feed. A daily sample was analyzed with a BioProfile FLEX2 cell and nutrient analyzer (Nova Biomedical, Massachusetts, USA) to measure the VCD, glucose, and lactate concentration and additional samples were analyzed after feed additions. Additional samples were collected at 74, 92, and 120 h after the first glucose addition for intracellular metabolite analysis. The collected analytics samples were quenched by 60% methanol with 0.8% ammonium formate at −20°C to slow cellular metabolism. A second set of 22 batches were run to validate the performance of the model trained on the first set; this run ended a day earlier due to a low working culture volume. Twelve batches were operated at 37°C for the whole batch, and 10 had their temperature reduced to 33°C on Day 3.

### 2.2 Calculation of Metabolite Transport Rates

The principle of mass conservation was applied to estimate the cells’ specific consumption rates for nutrients and specific secretion rates for byproducts. For a generic cell culture operation, this can be expressed as Equation (1) for all metabolites and process types where V is the bioreactor volume, [Met] is the metabolite concentration, *t* is time, *F* is the mass flowrate, *ρ* is density, and qMet is the specific metabolite transport rate. The subscripts *F*, *H*, and *B* indicate if the parameter belongs to a feed, harvest, and bleed stream, respectively.

(1) $$\frac{d\left(V\times \left[\text{Met}\right]\right)}{\text{dt}}=\frac{F_{F}}{\rho_{F}}\times \left[\text{Me}t_{F}\right]-\frac{F_{H}}{\rho_{H}}\times \left[\text{Me}t_{H}\right]-\frac{F_{B}}{\rho_{B}}\times \left[\text{Me}t_{B}\right]+\text{qMet}\times \left(\text{VCD}\times V\right),$$

(2) $$\text{qMet}\;=\frac{1}{\text{VCD}}\times \frac{d\left[\text{Met}\right]}{\text{dt}},$$

(3) $$\text{qMet}\;=\frac{1}{\text{VCD}}\times \frac{d\left[\text{pMet}\right]}{\text{dt}}.$$

For unfed batch processes, there is no mass flowing in or out of the bioreactor. The bioreactor volume was assumed to be constant and Equation (1) was solved for the metabolite transport rate to arrive at Equation (2). A pseudo metabolite concentration, [pMet], was created for the fed batch processes by accounting for the bolus feeds. For nutrients, the change in concentration of the metabolite due to a feed was subtracted from all subsequent metabolite concentrations. For metabolic byproducts, the change in concentration due to dilution from each feed was added to all subsequent metabolite concentrations. The metabolite transport rates were then determined from Equation (3).

**Figure 2:**
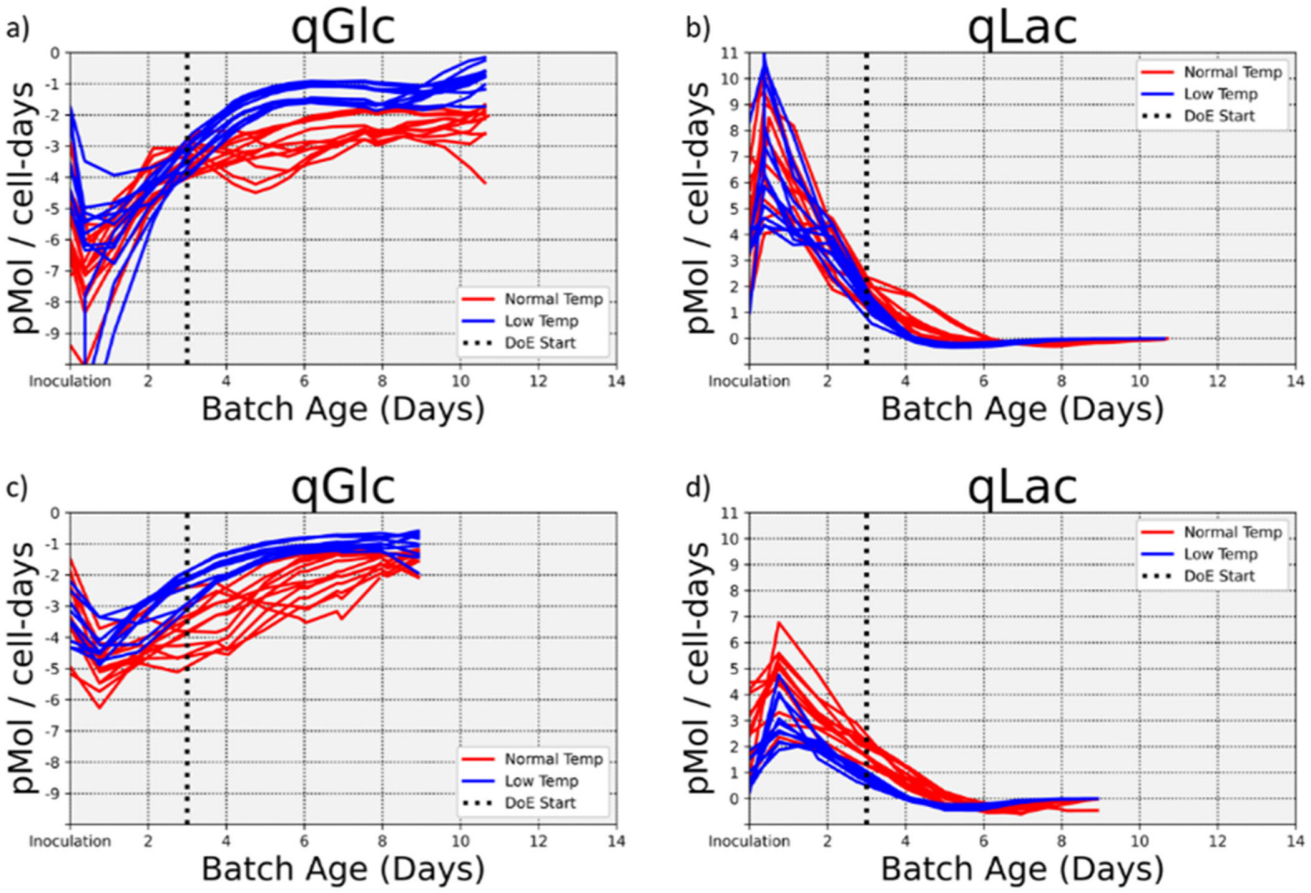
Metabolite transport rates. (a) and (b) show the glucose and lactate transport rates, respectively, for the 24 batches used for model training. (c) and (d) show the transport rates for the 22 batches used for model validation. After the temperature was reduced on Day 3 (indicated by the dotted line), the glucose consumption rate per cell and the lactate production rate per cell were reduced.

To smooth away noise in measurement and fill in missing values in the data, polynomials were fit to the time‐series metabolite concentrations. The smooth values of VCD were directly entered into the equation and the values of the derivative were found analytically from the polynomial coefficients that were fit to the metabolite concentrations. All calculations were performed using the cvxopt package (Diamond and Boyd 2016) in Python 3.6. The estimated metabolite transport rates are shown in Figure 2a,b.

### 2.3 Flux Balance Analysis (FBA)

FBA is a constraint‐based systems biology tool (Ivarsson et al. 2015) that can estimate the metabolic reaction rates, ***v***, inside a cell. Due to the pseudo steady‐state assumption in FBA, these rates must satisfy the reaction stoichiometries, ***S***, according to Equation (4). In this work, a previously published central carbon model for CHO cells (Pan et al. 2017) was used that had been updated to account for the ATP maintenance requirements identified in the genome scale model (Hefzi et al. 2016). In addition, to tailor the predicted reaction rates to the specific CHO cell culture processes under consideration, additional constraints of the form of Equation (5) were added for each of the metabolite transport rates at one time so that they must be within ±30% of the calculated values. The optimization problem in Equation (6) was then solved to find the set of reaction rates that maximize the creation of biomass and also satisfy the stoichiometry in Equation (4) and constraints of the form of (5). The evolution of metabolic activity was then found by repeating this process for each timepoint in all batches.

(4) S×v=0,

(5) $$\mathrm{lower}\;\mathrm{limi}t_{j}\leq v_{j}\leq \mathrm{upper}\;\mathrm{limi}t_{j},$$

(6) $$\begin{array}{c} \max\\ v \end{array}Z=v_{\mathrm{biomass}}.$$

All flux balance analysis models were fit using the COBRA toolbox (Heirendt et al. 2019) in MATLAB 2017b. One additional rate was calculated from the model fits: total rate of generating ATP was calculated from the reaction rates for all reactions that produce ATP.

### 2.4 Multivariate Analysis

Principal Component Analysis (PCA) is used to identify a set of orthogonal axes, referred to as principal components, that capture progressively smaller amounts of variance in the data (Price et al. 2006). Each observation of the original variables, ***X***, are projected onto these new axes. The values of each observation on these new axes are called scores, ***t***, and the orientation of the score space relative to the feature space are defined by loadings, ***p***, according to Equation (7) where ***p*** is selected to maximize the variance of ***t***. ***E*** is the residual matrix containing the portion of the variability in the original variables that is not captured by the *A* principal components extracted. Typically, only a few principal components are needed to describe most of the variability that arises from many features and, thus, reduces the complexity and makes the results easier to interpret. For all PCA models, the ***X*** block was comprised of the 196 reaction rates calculated by the FBA models and the total rate of ATP generation.

Partial Least Squares regression (PLS) is a tool for identifying the relationship between a set of features (Párta et al. 2014), ***X***, and another set of features, ***Y***, according to Equations (7) and (8). The loadings, ***p*** and ***q***, are selected to maximize the covariance between the scores, ***t*** and ***u***. ***F*** is the residual matrix containing the uncaptured variability of ***Y***. A Batch Evolution Model (BEM) (Alinaghi et al. 2022) is a special form of the PLS model where time is used as the ***Y*** feature to identify normal variations in ***X*** during the evolution of a biomanufacturing process. This allows control charts to be established for the scores, ***t***, that can be monitored to identify process changes. The ***X*** block remains the same as for the PCA models.

(7) $$X=\sum_{i=1}^{A}t_{i}p_{i}^{T}+E,$$

(8) $$Y=\sum_{i=1}^{A}u_{i}q_{i}^{T}+F.$$

In all models, the metabolic reaction rates, *v*, were scaled to unit variance before analysis. All models were fit using SIMCA 18 (Berry et al. 2015) (Sartorius, DE).

## 3 Results

The metabolite transport rates are shown in Figure 2. The glucose and lactate transport rates are shown for the first batch of 24 reactors in Figure 2a,b, respectively. The values are shown for the second batch of 22 reactors in Figure 2c,d for glucose and lactate, respectively. There are differences in the glucose consumption rate after Day 3 when the temperature shift was induced. The reduced temperature led to a reduced glucose consumption rate per cell. The reduced temperature also was associated with a reduced production of lactate. Taken together, the data were consistent with a slowdown in the overall metabolic activity with the reduced temperature.

**Figure 3:**
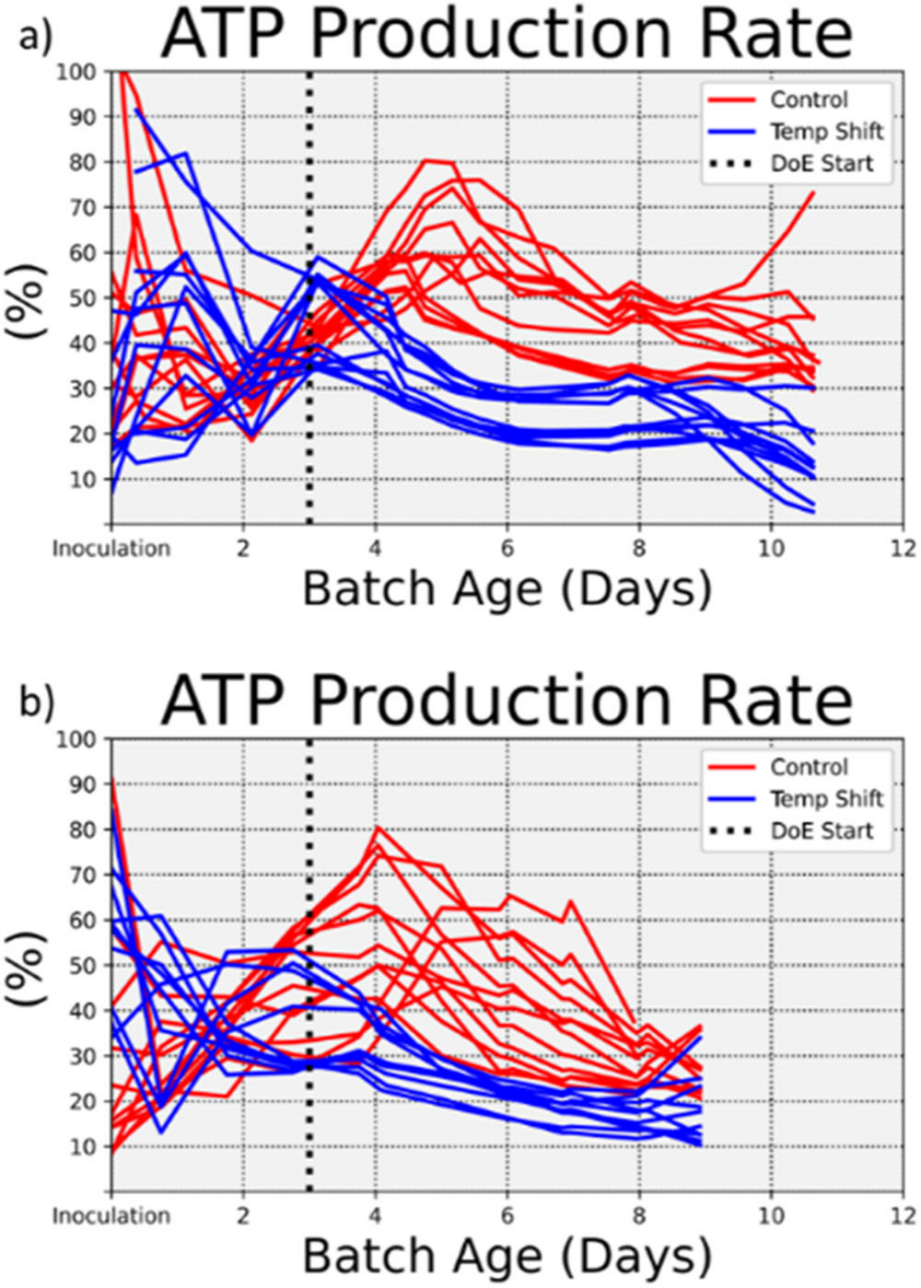
Evolution of total ATP generation rate. (a) and (b) show the ATP production rates for the training data and model validation data, respectively, that were estimated by the flux balance analysis models. After the temperature was reduced on day 3, the rate of ATP being generated per cell was reduced.

The total rate of generated ATP is shown in Figure 3a,b for the first and second batch, respectively. Due to the pseudo‐state hypothesis, each individual data point represents a single FBA model fit that was fit independently of all others. There were an additional 196 reaction rates that were calculated for each ATP generation rate shown here. Despite the pseudo steady state hypothesis, the evolution of internal metabolism was captured by the individual steady‐state model fits analogously to the way the dynamic evolution of a movie scene is captured by individual still frames.

The rate of ATP generated in Figure 3 showed that the deliberately reduced temperature on Day 3 for selected batches had a noticeable effect on internal cellular metabolism. The reduced temperature led to less ATP being generated.

**Figure 4:**
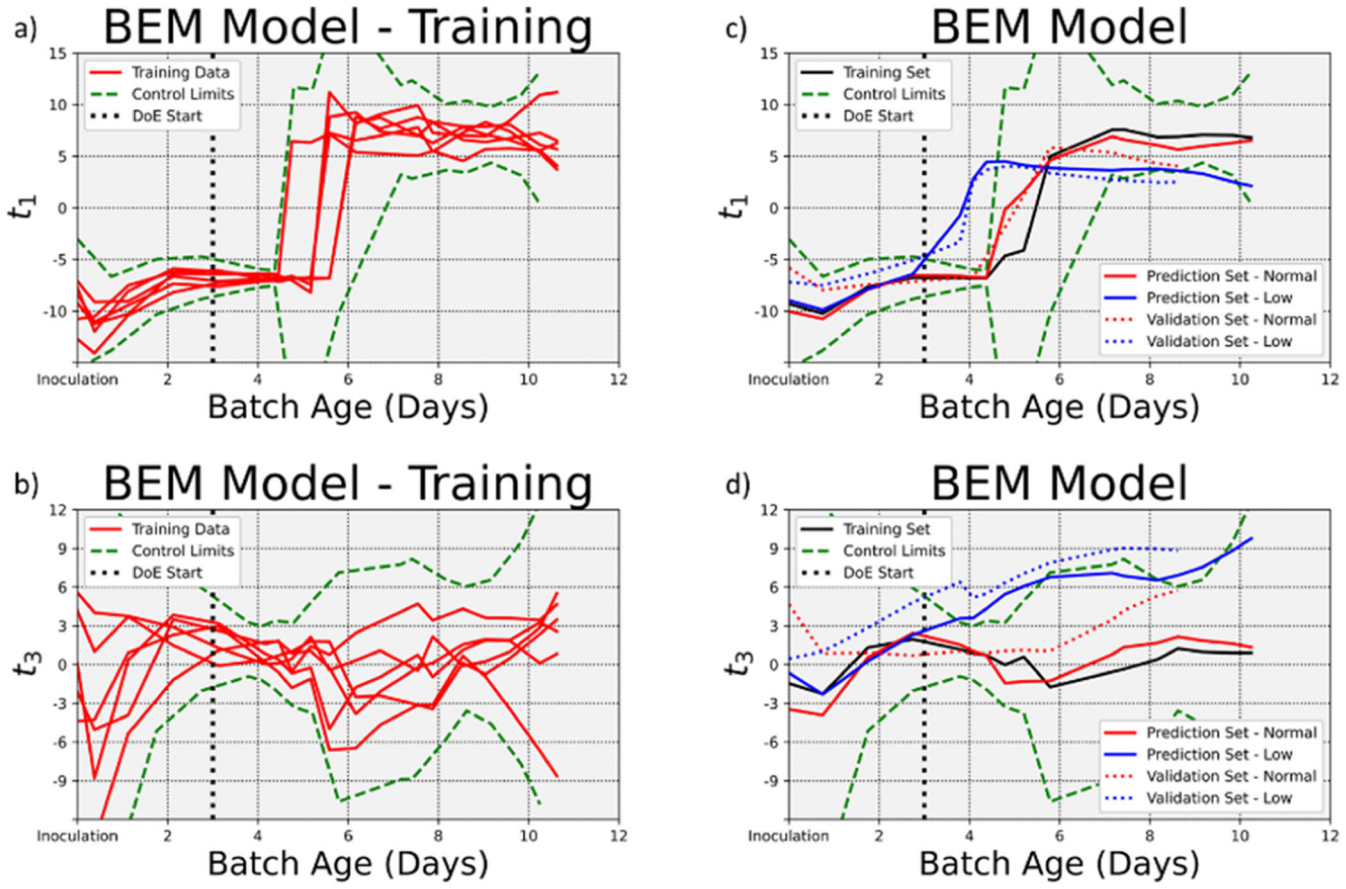
Batch Evolution Model (BEM) control charts. (a) and (b) show the construction of the golden batch control charts on the first and third principal components, respectively, from the six batches operated normally for model training. The loadings generated during model training were used to project the remaining data into the score space, shown in (c) and (d) for the first and third principal components, respectively. The prediction set and validation set reactors operated normally, shown in red, behaved similarly to the batches operated normally for model training. The reactors operated with a temperature shift after Day 3, shown in blue, quickly go outside of the control limits after Day 3. This indicates that the macroscopic process change can be detected from changes in the internal cellular metabolism.

We trained a Batch Evolution Model (BEM) with six of the reactors operated normally. The resulting control charts, shown in Figure 4a,b for principal component 1 and 3, respectively, were derived from the ±3 standard deviation limits around the average score value, shown in green in Figure 4. Collectively, these two control charts define the “golden batch.” The control chart for score 2 is not shown because the variability in the original data captured by the second principal component was not related to the temperature shift. The average of these 6 reactors are shown in black in Figure 4c,d for the first and third principal components, respectively. The 18 reactors from this batch that were not used for model training, collectively referred to as the prediction set, were projected into the score space using the BEM loadings. The average scores of the 6 remaining reactors that were operated normally are shown in red, and the average scores for the 12 reactors that were operated with a low temperature are shown in blue in Figure 4c,d. The reactors operated normally behaved similarly to the batches used for model training, the solid red line tracks the black line closely throughout the cell culture on both principal components. By contrast, the reactors whose temperature were reduced on Day 3 do not behave similarly. The solid blue line tracks the red and black line up until Day 3 before diverging after Day 3. These lower temperature reactors went outside of the control limits set by the training data after Day 3 even though temperature was not directly included in the model.

To verify that these results could hold true for future batch processes, a second batch of validation runs was performed that comprised of 12 reactors operated normally and 10 reactors operated with a low temperature after day 3. The BEM loadings were used to project the average of these batches into the score space. The resulting average is shown with a dotted red line for the normal batches and a dotted blue line for the batches operated with a low temperature in Figure 4c,d. Here again, the batches operated normally do not go outside of the BEM control limits and the batches operated with a low temperature exit the control limits.

**Figure 5:**
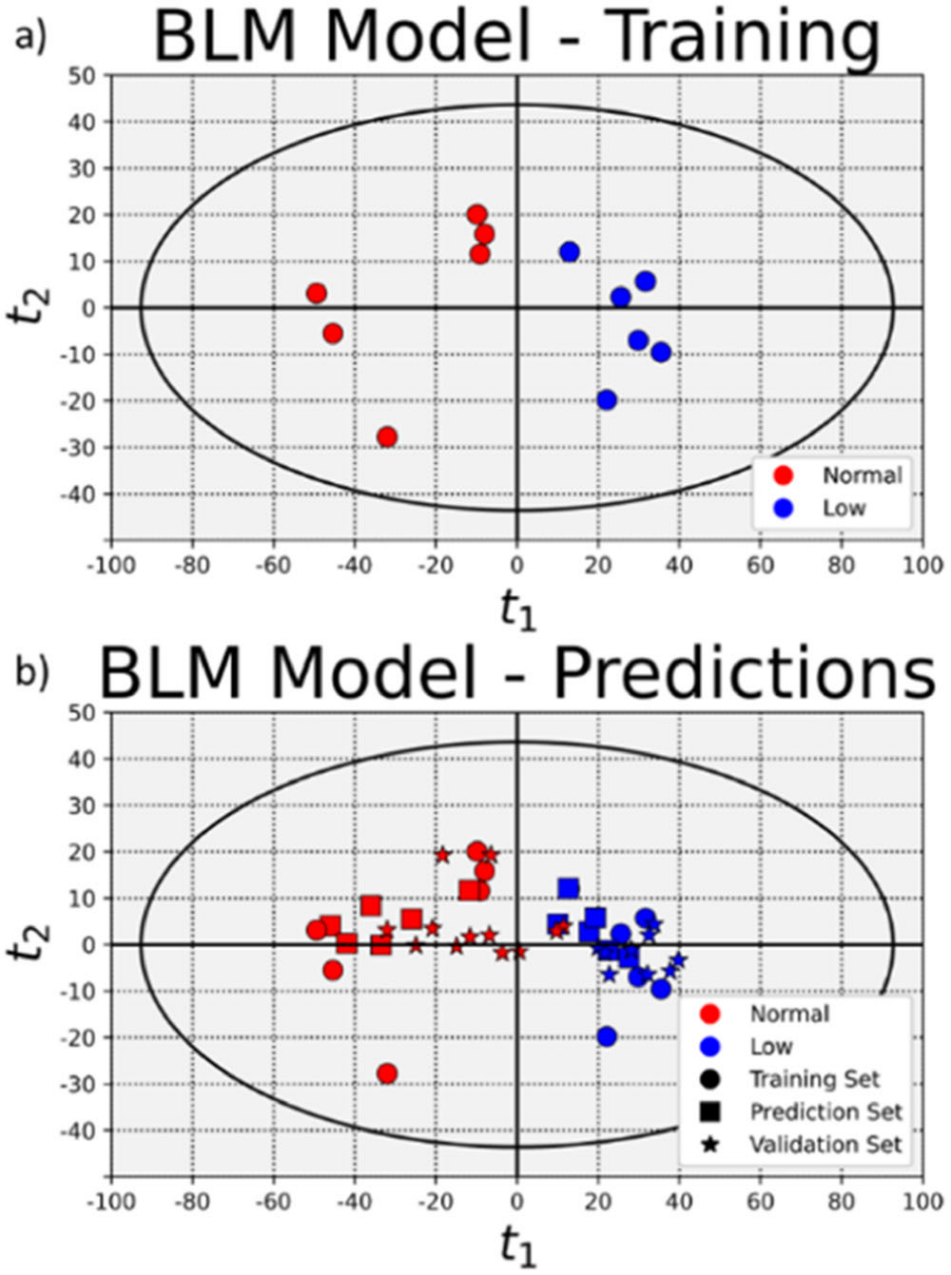
Batch level models. (a) shows the score space generated from six batches operated normally (red) and six batches operated with a temperature shift (blue). The loadings were used to project the remaining data into the score space, shown in (b). Batches that project in the left half are classified as having been operated at normal conditions, and batches that project into the right half are classified as having been operated with a temperature shift after Day 3. 15 of 18 batches operated normally in the prediction and validation sets were classified correctly. 16 of 16 batches operated with a temperature shift were classified correctly.

A batch level model (BLM) was constructed by performing a principal component analysis on the data where each combination of time‐reaction rate was treated as a separate variable for each of 12 reactors in the training data; 6 reactors that were operated at normal conditions and 6 that were operated with a temperature shift. The resulting score space is shown in Figure 5a; normal reactors are shown as red circles and reactors operated with a low temperature after Day 3 are shown as blue circles. The data showed that BLM could be used to classify future batches as having been normal or having experienced a low temperature process change. Batches that project into the left half of the score space could be classified as a batch operating at normal conditions and batches that project into the right half could be classified as those that were operated with a low temperature after day 3. The loadings were used to project the 12 remaining reactors from the first batch into the same score space and are shown as in Figure 5b as squares. Our results show that of the 12 reactors projected into the score space, all of them were accurately classified. Data from the additional 22 reactors in the second batch were analyzed and the result of projecting these 22 batches are shown as stars in Figure 5b. Nine of 12 batches operated normally were accurately classified as being operated normally. Every batch operated with a low temperature was accurately classified as being operated with a low temperature. In total, there was a 0% error rate for classifying the batches that were operated with a low temperature. There was a 12.5% error rate for classifying the batches that were operated normally.

## 4 Discussion

While genome scale representations of the reaction stoichiometries (***S***) are available for CHO cells, the limited number of metabolites that are measurable with currently available PAT on the manufacturing floor preclude its use for monitoring the evolution of cellular metabolism. Thus, modeling the cellular metabolism could allow additional insight into quality impacts without needing to measure all potentially important metabolites in the cellular media. Higher nutrient availability translates to larger fluxes in the energy producing pathways, and higher byproduct availability reduces the fluxes in some energy producing pathways. Further, the multivariate analysis models are used to characterize the variability in the data. By scaling to unit variance before building these models, any inaccuracies in the magnitude of the fluxes are dropped from the analysis.

Overall, the current application of the CMP driven tool presented here was shown to be useful for the process monitored in this work and may be useful for other biomanufacturing processes. For example, the energy production capacity of the cells in the glycolysis pathway is an important metric for vaccine production processes where the virus hijacks the glycolysis pathway for its own purposes (Akram 2013).

The work presented here shows that there are opportunities to better understand the biomanufacturing processes with regard to metabolic activity to supplement contemporary measurements of macroscopic process conditions. The metabolic activity that produces in‐spec product at lab scale should be comparable on average to metabolic activity at the manufacturing scale (Gargalo et al. 2020). Notably, reaching the same metabolic activity found at the smaller scale throughout a 5000 L reactor may not be possible throughout the entire volume because of larger gradients in factors like sheer stress or temperature (Limberg et al. 2017). Therefore, we anticipate the possibility of manufacturers supporting their development programs by generating a metabolic design space at a bench scale and using this information to support process scale‐up. Along these lines, manufacturers may be able to bring their product to market faster by using the metabolic design space at the bench scale as a target for metabolic behavior at production scale.

Thus, we propose CMP monitoring may result in savings during process development (Kasemiire et al. 2021; Metze et al. 2020; Niklas and Heinzle 2012; Sha et al. 2018). Unfed batch and fed‐batch processes could be used for process development to identify the metabolic conditions that lead to the most productive cells creating consistent high‐quality product. With those optimal conditions, a perfusion process may be developed to recreate a similar metabolic state. The metabolic conditions identified in the unfed batch or fed‐batch process could be used as a target for the metabolic conditions that should be experimentally justified in the perfusion process. Further, we anticipate that it could be possible to detect poor quality product from a perfusion process using the internal metabolic state to support that decision.

## 5 Conclusion

Here, internal cellular metabolic reaction rates have been shown to be reproducibly estimated using Flux Balance Analysis. Further, by monitoring these reaction rates with multivariate models it was possible to detect macroscopic process changes from only internal metabolic reaction rates. Batch Evolution Model control charts were shown to accurately detect when a temperature shift occurred from changes in the estimated internal metabolic reaction rates. In addition, Batch Level Models were shown to be useful for creating a metabolic fingerprint that can be used to identify the macroscopic process conditions of a process after the process was ended.

## Author Contributions

**Nicholas Trunfio:** conceptualization, formal analysis, investigation, methodology, project administration, resources, software, supervision, validation, visualization, writing–original draft, writing–review and editing. **David N. Powers:** conceptualization, investigation, methodology, project administration, resources, supervision; validation, visualization, writing–original draft, writing–review and editing. **Cyrus Agarabi:** conceptualization, project administration, resources, supervision, methodology, writing–review and editing. **Roberta King:** supervision, methodology, writing–review and editing. **Casey l. Kohnhorst:** methodology; resources; writing–review and editing. **Erica J. Fratz‐Berilla:** methodology; resources; writing–review and editing. **Xin Bush:** formal analysis; investigation; methodology; project administration; validation; visualization; writing–review and editing.

## Disclaimer

This publication reflects the views of the author and should not be construed to represent FDA's views or policies. Certain commercial equipment, instruments, or materials are identified in this paper to foster understanding. Such identification does not imply recommendation or endorsement by the FDA.

## Data Availability

All the relevant data are within this publication.
